# En respuesta a: El paciente con estenosis aórtica severa hospitalizado

**DOI:** 10.47487/apcyccv.v6i4.566

**Published:** 2025-12-29

**Authors:** José de Jesús Bohórquez-Rivero, Paula Andrea Parra-Sánchez, Angela María Roa-Bocanegra

**Affiliations:** 1 Universidad de Cartagena, Cartagena, Colombia. Universidad de Cartagena Universidad de Cartagena Cartagena Colombia; 2 Universidad del Sinú Elías Bechara Zainúm, Cartagena, Colombia. Universidad del Sinú Universidad del Sinú Elías Bechara Zainúm Cartagena Colombia; 3 Universidad Autónoma de Bucaramanga, Santander, Colombia. Universidad Autónoma de Bucaramanga Universidad Autónoma de Bucaramanga Santander Colombia; 4 Universidad El Bosque, Bogotá, Colombia. Universidad El Bosque Universidad El Bosque Bogotá Colombia

*Sr. Editor*

Hemos leído con gran interés la carta al editor titulada «El paciente con estenosis aórtica severa hospitalizado», publicada en el último volumen de la revista *Archivos Peruanos de Cardiología y Cirugía Cardiovascular*^1^, en la cual el autor describe aspectos relevantes del abordaje de esta valvulopatía, en su mayoría basados en las recomendaciones de las guías del American College of Cardiology/American Heart Association (ACC/AHA) ^2^ y en la clasificación propuesta por Généraux, *et al*. ^3^. Sin embargo, en aras de potenciar el conocimiento en la aproximación a esta entidad, consideramos pertinente ampliar la información con base en las guías recientemente publicadas por la European Society of Cardiology/European Association for Cardio-Thoracic Surgery (ESC/EACTS) en 2025 ^4^*.*

Dichas guías clasifican la gravedad de la estenosis aórtica (EAo) de acuerdo con parámetros ecocardiográficos como el gradiente medio (GM) de presión (siendo el componente más robusto para su valoración), la velocidad máxima transvalvular (Vmax) y el área valvular aórtica (AVA) efectiva. Aunque el AVA representa, en teoría, el indicador ideal, su cálculo se ve limitado por numerosas variables técnicas, por lo que la interpretación integrada de los tres parámetros resulta esencial.

Las guías proponen, además, categorizar la EAo según el estado de flujo, determinado por el volumen sistólico indexado (VSi), especialmente en escenarios donde los parámetros ecocardiográficos son discordantes. De manera convencional, un VSi inferior a 35 mL/m² define una situación de bajo flujo.

A diferencia de las guías ACC/AHA ^2^, que subdividen la EAo severa en fenotipos C1-C2 (asintomáticos) y D1-D3 (sintomáticos) de acuerdo con la función ventricular izquierda (FEVI), el estado de flujo y la presencia de síntomas, las guías ESC/EACTS 2025 no adoptan esta clasificación alfanumérica. Tampoco incorporan los términos propuestos por Généraux, *et al*. *(3)* de síndrome valvular estable, progresivo o agudo. En su lugar, describen los distintos escenarios fisiopatológicos según la integración de parámetros hemodinámicos.

En este contexto, la EAo de alto gradiente, definida por un GM ≥40 mmHg, una Vmax ≥4,0 m/s y un AVA ≤1 cm² (o AVA indexada ≤0,6 cm²/m²), se considera severa, independientemente de la función ventricular izquierda o de las condiciones de flujo. No obstante, la ESC/EACTS enfatiza la existencia de formas discordantes de EAo, cuyo reconocimiento es clave para evitar errores diagnósticos y terapéuticos.

Entre estas se incluyen:

- EAo de bajo flujo y bajo gradiente con FEVI reducida (GM<40 mmHg, AVA ≤1 cm², VSi ≤35 mL/m², FEVI <50%).
- EAo de bajo flujo y bajo gradiente con FEVI conservada (GM <40 mmHg, AVA ≤1 cm², VSi ≤35 mL/m², FEVI ≥50%).
- EAo de flujo normal y bajo gradiente con FEVI conservada (GM <40 mmHg, AVA ≤1 cm², VSi >35 mL/m², FEVI ≥50%).
- EAo discordante de alto gradiente, con GM ≥40 mmHg, pero AVA >1 cm².

Los pacientes con EAo de flujo normal y bajo gradiente suelen presentar estenosis moderada, mientras que las formas discordantes de alto gradiente se consideran graves, salvo que exista un estado de alto flujo reversible (anemia, hipertiroidismo o fístulas arteriovenosas).

Para el abordaje diagnóstico de las formas discordantes se recomienda una evaluación escalonada y multimodal. En pacientes con EAo de bajo flujo y bajo gradiente con FEVI reducida, la ecocardiografía de estrés con dobutamina puede distinguir entre una EAo pseudosevera y una EAo verdaderamente severa, siempre que exista reserva contráctil, definida como un incremento del volumen sistólico ≥20%.

De forma complementaria, la tomografía computarizada cardíaca con cuantificación de calcio valvular aórtico (puntuación de Agatston) se consolida como una herramienta diagnóstica de gran valor. Valores superiores a 2000 unidades Agatston (UA) en hombres y 1200 en mujeres son altamente sugestivos de EAo severa, mientras que cifras inferiores a 1600 y 800 UA, respectivamente, hacen improbable dicha condición. Esta técnica resulta particularmente útil cuando la ecocardiografía es discordante cuando existen factores que limitan la calcificación valvular (ejemplos: válvula aórtica bicúspide, amiloidosis cardíaca o estenosis fibrosa posreumática o inducida por radiación).

Asimismo, las guías recomiendan considerar parámetros complementarios, como el índice adimensional o relación de velocidades (VTI del TSVI/VTI del jet aórtico), en el que un valor <0,25 sugiere una EAo grave, y el *strain* longitudinal global (SLG), útil tanto para la estratificación pronóstica como para identificar daño miocárdico extravalvular. Un SLG ≤−15% se asocia con mayor riesgo de deterioro clínico o mortalidad prematura en pacientes asintomáticos.

La impedancia valvuloarterial emerge como un marcador pronóstico de desenlaces adversos antes y después del reemplazo valvular. Por otra parte, los péptidos natriuréticos sirven para identificar a los pacientes asintomáticos con EAo grave de alto riesgo, que podrían beneficiarse de una intervención temprana.

De igual modo, la prueba de esfuerzo y la ecocardiografía de ejercicio permiten desenmascarar síntomas y detectar intolerancia hemodinámica, particularmente cuando la presión arterial desciende >20 mmHg, herramientas útiles para la estratificación del riesgo y la toma de decisiones en EAo grave asintomática. La prueba de ejercicio cardiopulmonar, eventualmente combinada con ecocardiografía, puede diferenciar entre limitación cardíaca, pulmonar o descondicionamiento físico.

Por último, la resonancia magnética cardíaca se destaca en la evaluación del remodelado ventricular y la cuantificación de fibrosis miocárdica, ambos factores pronósticos de eventos adversos.

**Figura 1:**
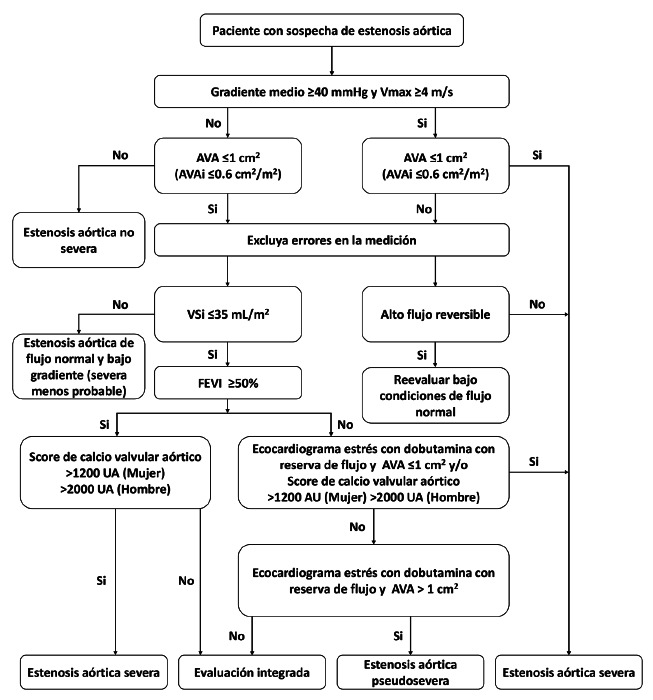
Evaluación imagenológica integral de pacientes con EAo ^4^.

En síntesis, las guías ESC/EACTS 2025 refuerzan una visión más integradora y fisiopatológica de la EAo, destacando la importancia del análisis combinado de parámetros clínicos, bioquímicos y hemodinámicos, así como la utilización de métodos de imagen avanzados para caracterizar mejor dicha entidad. Este enfoque multidimensional facilita un diagnóstico más preciso y una selección más adecuada de los pacientes candidatos a intervención, contribuyendo así a mejorar los desenlaces clínicos y la toma de decisiones terapéuticas**. Las**Figuras 1**y**2 ilustran el abordaje diagnóstico y terapéutico propuesto en estas directrices.

Vmax: velocidad máxima. AVAi: área valvular aórtica indexada. VSi: volumen sistólico indexado. FEVI: fracción de eyección del ventrículo izquierdo.

**Figura 2:**
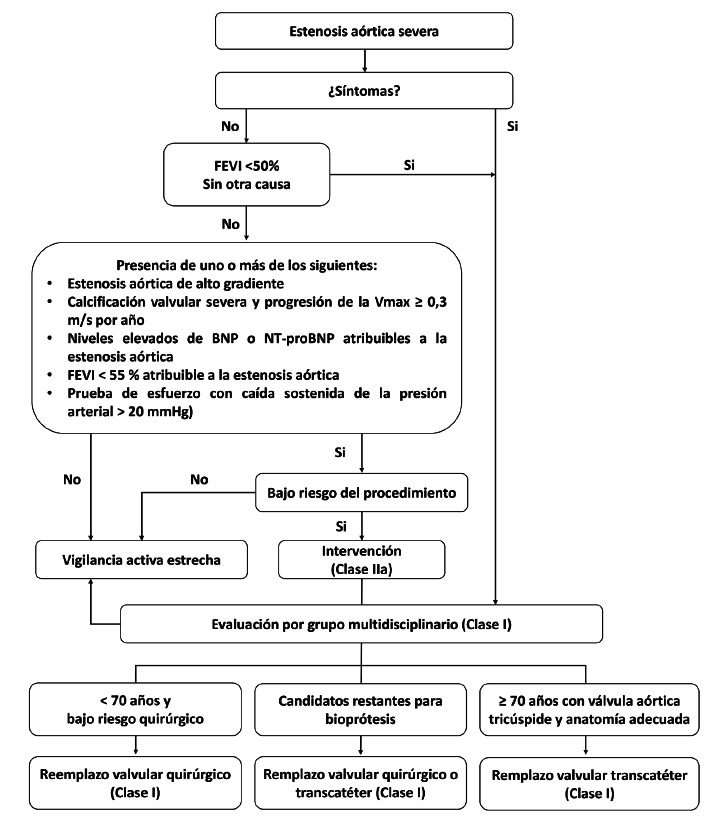
Manejo de los pacientes con EAo severa ^4^.

Vmax: velocidad máxima. FEVI: fracción de eyección del ventrículo izquierdo. BNP: péptido natriurético tipo B. NT-proBNP: péptido natriurético tipo B N-terminal.
